# Targeted Destruction of Photosensitive Retinal Ganglion Cells with a Saporin Conjugate Alters the Effects of Light on Mouse Circadian Rhythms

**DOI:** 10.1371/journal.pone.0003153

**Published:** 2008-09-05

**Authors:** Didem Göz, Keith Studholme, Douglas A. Lappi, Mark D. Rollag, Ignacio Provencio, Lawrence P. Morin

**Affiliations:** 1 Department of Biology, University of Virginia, Charlottesville, Virginia, United States of America; 2 Department of Psychiatry, Stony Brook University, Stony Brook, New York, United States of America; 3 Program in Neuroscience, Medical Center, Stony Brook University, Stony Brook, New York, United States of America; 4 Advanced Targeting Systems, San Diego, California, United States of America; University of Southern California, United States of America

## Abstract

Non-image related responses to light, such as the synchronization of circadian rhythms to the day/night cycle, are mediated by classical rod/cone photoreceptors and by a small subset of retinal ganglion cells that are intrinsically photosensitive, expressing the photopigment, melanopsin. This raises the possibility that the melanopsin cells may be serving as a conduit for photic information detected by the rods and/or cones. To test this idea, we developed a specific immunotoxin consisting of an anti-melanopsin antibody conjugated to the ribosome-inactivating protein, saporin. Intravitreal injection of this immunotoxin results in targeted destruction of melanopsin cells. We find that the specific loss of these cells in the adult mouse retina alters the effects of light on circadian rhythms. In particular, the photosensitivity of the circadian system is significantly attenuated. A subset of animals becomes non-responsive to the light/dark cycle, a characteristic previously observed in mice lacking rods, cones, and functional melanopsin cells. Mice lacking melanopsin cells are also unable to show light induced negative masking, a phenomenon known to be mediated by such cells, but both visual cliff and light/dark preference responses are normal. These data suggest that cells containing melanopsin do indeed function as a conduit for rod and/or cone information for certain non-image forming visual responses. Furthermore, we have developed a technique to specifically ablate melanopsin cells in the fully developed adult retina. This approach can be applied to any species subject to the existence of appropriate anti-melanopsin antibodies.

## Introduction

The hypothalamic suprachiasmatic nuclei (SCN) comprise the primary circadian pacemaker in mammals and are responsible for the generation of nearly all daily rhythms in physiology and behavior. Timing of such rhythms is set by the daily photoperiod. The retinohypothalamic tract (RHT) arises from a subset of retinal ganglion cells and conveys photic information to the SCN through the optic nerve. Most of these retinal ganglion cells express melanopsin photopigment (Opn4) and are intrinsically photosensitive [1], [2]. These intrinsically photosensitive retinal ganglion cells (ipRGCs) project to numerous brain regions, in addition to the SCN [3].

Mice lacking either the classical photoreceptors (rods and cones) [4], [5] or melanopsin photopigment [6]–[8] exhibit relatively normal entrainment of circadian rhythms to the light∶dark photoperiod, masking, and constriction of the pupil in response to ocular illumination. However, the absence of both classical photoreceptors and melanopsin eliminates all three responses to light [9], [10].

The purpose of these studies was to determine whether photic information received by classical photoreceptors requires the presence of ipRGCs in order to modify non-image-forming visual responses. Toward this end, we developed a saporin-based immunotoxin (UF008/SAP) that specifically ablates ipRGCs in the fully-differentiated adult retina. Importantly, this approach can be modified to target melanopsin-expressing cells in any animal model, even those genetically intractable, provided that the appropriate targeting antibodies are available.

## Results

### Specific Targeting of ipRGCs

**Figure 1:**
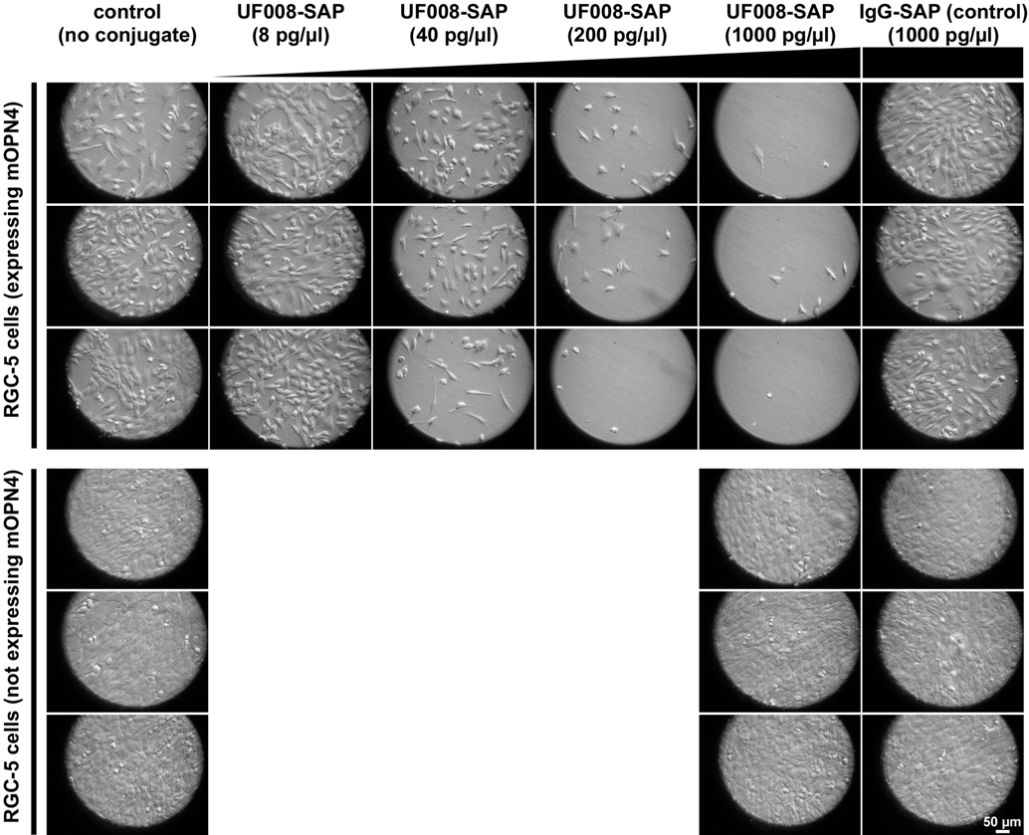
A saporin/anti-melanopsin(UF008) conjugate destroys cultured RGC-5 cells in a dose-dependent manner. Cultures of RGC-5 cells, either stably expressing or not expressing mouse melanopsin, were exposed to a saporin conjugate (UF008/SAP) for 4 days. The concentrations of conjugates are shown above each column and the experiments were done in triplicate. Each panel represents a randomly selected field from a single well.

In order to show specific ablation of melanopsin expressing cells, RGC-5 cells stably expressing melanopsin were exposed to the UF008/SAP conjugate. After 4 days of exposure, the cells were killed in a dose-dependent manner. The highest concentration of UF008/SAP (1000 pg UF008/SAP /µl) caused maximum cell death whereas the same concentration of the non-immunized IgG/SAP conjugate did not cause any cell death (Figure 1). Also, RGC-5 cells that do not express melanopsin were not affected by the UF008/SAP conjugate, even at the highest dose tested.

**Figure 2:**
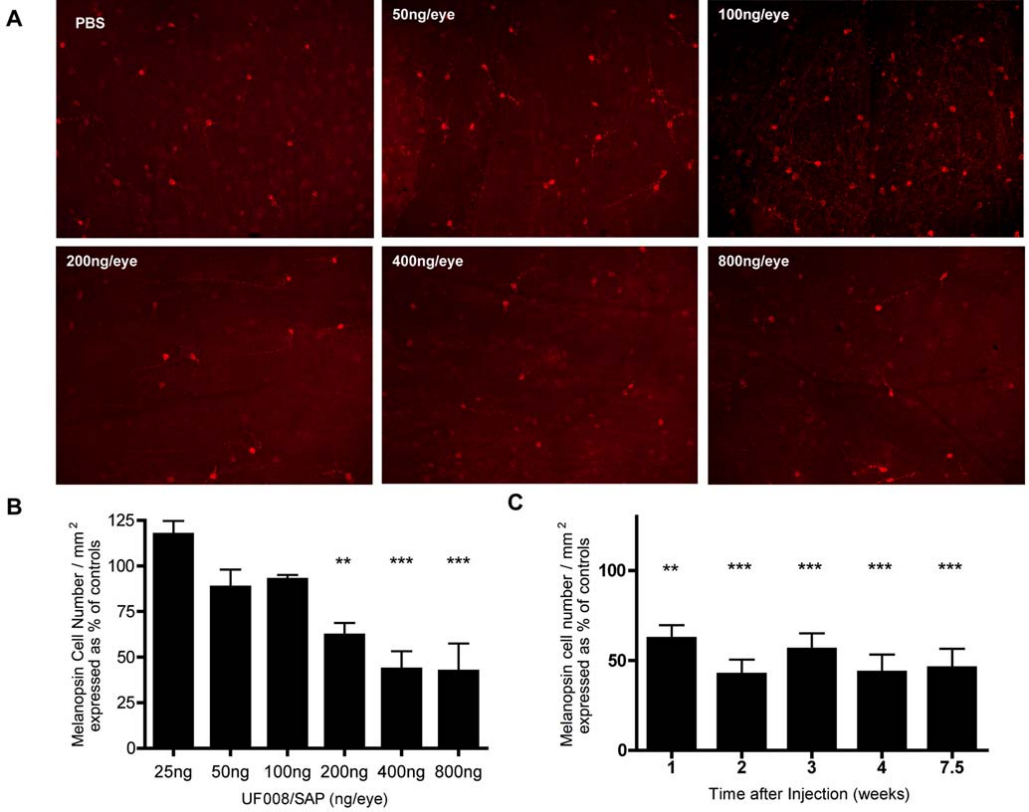
*In vivo* injection of UF008/SAP rapidly destroys ipRGCs in a dose-dependent manner. UF008/SAP-dependent killing of ipRGCs in adult C57BL/6J mouse retina. A) Selected retinal flat-mount images (10× magnification) of PBS and UF008/SAP injected eyes from each dose group. B) Left eyes of mice (n = 5 per dose group) were injected with increasing doses of the UF008/SAP conjugate (25, 50, 100, 200, 400 and 800 ng/eye). Right eyes were injected with PBS to serve as controls. (1-way ANOVA with Tukey's Multiple Comparison Test, ** p<0.01, *** p<0.001). C) Mice (n = 4 per time group) were bilaterally injected with 400 ng of UF008/SAP conjugate per eye or 400 ng/eye of IgG/SAP (saporin conjugated to a “nonsense” rabbit IgG). At one week post-injection, the remaining number of melanopsin cells in the UF008/SAP injected retinas was significantly less than the controls, but the maximum melanopsin cell death was achieved at 2 weeks post-injection (1-way ANOVA with Tukey's Multiple Comparison Test, * p<0.05, *** p<0.001). The results for each experimental group in B and C were normalized relative to the data from the contralateral control eyes.

**Figure 3:**
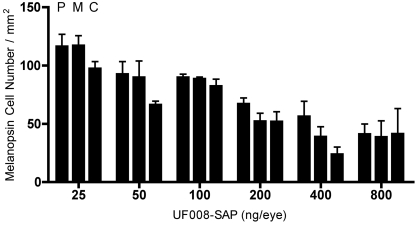
Destruction of ipRGCs by UF008/SAP depends on dose, but not on location within the retina. Ablation of ipRGCs by UF008/SAP is dose-dependent, but not related to retinal eccentricity. Each triplet of bars represents data from “peripheral” (P), ”middle” (M), and “central” (C) fields, respectively. (2-way ANOVA with Bonferroni post-tests).

Injection of the UF008/SAP conjugate into the vitreous of adult C57BL/6J mouse eyes killed ipRGCs in a dose-dependent manner (Figure 2A and B), the curve becoming asymptotic at approximately 400 ng/eye. All control retinas from various dose groups had similar melanopsin cell densities and were combined. Relative to the average control retina, about 57% of melanopsin cells were killed by 400 ng UF008/SAP per eye which was not significantly different than the cell death achieved with the highest dose group (800 ng/eye). Representative images from each dose group (Figure 2A) show the decreasing number of immunopositive cells in retinas exposed to increasing UF008/SAP amounts. A time course study showed that maximal melanopsin cell death occurs about 2 weeks after 400 ng UF008-SAP injection (Figure 2C). Each group was significantly different from controls. Retinas of the 4 week group that received 400 ng/eye UF008/SAP were analyzed for regional differences in the efficacy of the conjugate. Analysis of peripheral to central regional variation in UF008/SAP-dependent melanopsin cell death in the mouse retina revealed that the density of remaining cells was uniform indicating no regional differences (Figure 3).

**Figure 4:**
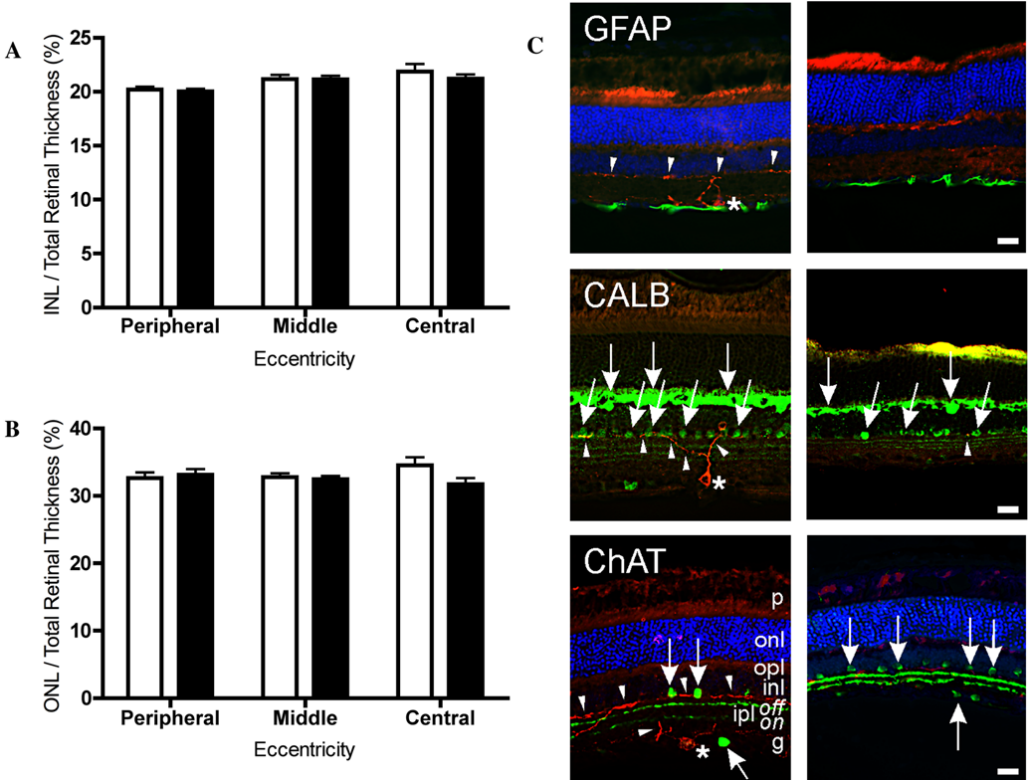
UF008/SAP kills ipRGCs without apparent damage to general retinal morphology. UF008/SAP does not induce changes in gross retinal morphology. Eyes injected with 800 ng/eye of UF008/SAP conjugate did not differ ( 2-way ANOVA with Bonferroni post-test) from the PBS-injected control eyes in their relative (A) ONL or (B) INL thickness. (open bars, control eyes; black bars, UF008/SAP-injected). C) Cryostat sections through control retina (left column) and through UF008/SAP treated retina (right column). Red label (all images) - Melanopsin immunoreactive (IR) cells (*) and processes (arrowheads); Green label (Upper Row) - GFAP-IR glia at the base of the ganglion cell layer; Green label (Middle Row) - CALB-IR cells (arrows) in the outer and inner nuclear layers; Green label (Bottom row) - ChAT-IR amacrine cells (arrows) in the inner nuclear and ganglion cell layers between which are the two ChAT-IR terminal zones of the inner plexiform layer. Blue label - The nuclear stain, DAPI, most clearly reveals cells in the outer nuclear layer of the upper and lower images. Note the absence of melanopsin-IR staining in treated retinas (right column). Abbreviations of retinal layers: g – ganglion cell; inl – inner nuclear; ipl – inner plexiform (on and off sublayers); onl – outer nuclear; opl – outer plexiform; p – photoreceptor. Bar = 20 µm.

There are various ways of assessing retinal health, but relative thickness of the outer nuclear layer (ONL) has been used previously as a direct measurement of the degree of photoreceptor cell death [11], [12]. Figure 4A and B shows that neither relative ONL nor relative inner nuclear layer (INL) thickness of retinas from mice receiving 800 ng/eye UF008/SAP differed from corresponding measurements from PBS-injected retinas. Because there was no significant effect of the highest dose, no effects were expected with the lower doses.

Exposure of the retina to UF008/SAP does not affect its tissue morphology, as non-melanopsin cells remain intact. Cellular analysis in Figure 4C confirms that UF008/SAP (400 ng/eye) causes the loss of intrinsically photoreceptive ganglion cells (ipRGCs), but does not appear to alter other structural characteristics of the mouse retina. A normal distribution of glial fibrillary acid protein (GFAP; labels Muller cells), calbindin- (CALB; labels horizontal cells) and choline acetyltransferase (ChAT; labels cholinergic amacrine cells) immunoreactivity was observed in both control and UF008/SAP injected eyes.

**Figure 5:**
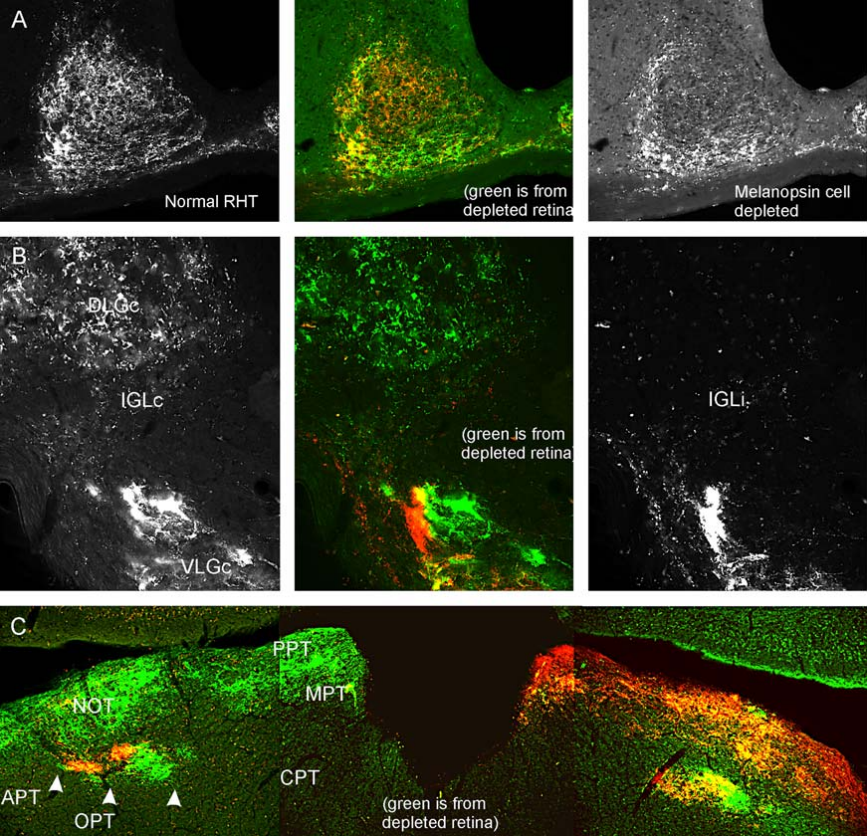
UF008/SAP greatly reduces retinal projections to the suprachiasmatic nucleus and intergeniculate leaflet. CT-B tracing of RHT projections remaining after intravitreal UF008/SAP injection shows a terminal field densest along the lateral, ventrolateral and ventral SCN border, but greatly reduced in, or absent from, the dorso-central SCN. B) In the IGL, the contralateral projection from the UF008/SAP-injected retina is largely absent. C) There are no readily apparent differences with respect to the remaining retinal innervation of the OPT. The red label in all parts of the figure identifies terminals from the contralateral, undamaged retina. Abbreviations: APT – anterior pretectal n.; CPT – commissural pretectal n.; DLGc – dorsal lateral geniculate n;, contralateral; IGLc – intergeniculate leaflet, contralateral; IGLi – intergeniculate leaflet, ipsilateral; MPT – medial pretectal n.; NOT – nucleus of the optic tract; PPT – posterior pretectal n.; RHT – retinohypothalamic tract; VLGc – ventral lateral geniculate n., contralateral. (Ipsi- and contra- are referenced with respect to the injected with UF008/SAP).

Anatomical evaluation of the RHT terminal plexus in the SCN was performed in mice that had received unilateral UF008/SAP injections. The lesioned eye was given an intra-vitreous injection of cholera toxin subunit B (CT-B) conjugated to Alexa488 and the intact eye was injected with CT-B/Alexa594 conjugate. Ipsi- and contralateral retinas contribute equally to mouse SCN innervation [13], allowing the contributions of lesioned and intact retinas to be compared. Ablation of ipRGCs yielded an unexpected topography of remaining SCN retinal innervation. Fluorophore-conjugated CT-B labeled a dense corona of terminals along the lateral and ventral SCN border, with very sparse innervation centrally and medially. The normal retinal input occupied the central/medial area, as well as overlapping the more lateral terminal corona (Figure 5).

### Behavioral Experiments

**Figure 6:**
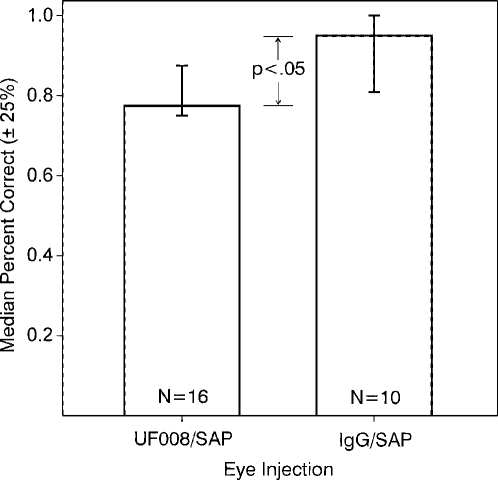
UF008/SAP treatment has little effect on visual cliff performance. Both UF008/SAP and IgG/SAP injected groups perform quite accurately in the visual cliff test, but there is a small deficit in the UF008/SAP treated group (p<0.05, Mann-Whitney test).

Having revealed there are no significant morphological differences between retinas dissected from UF008/SAP injected and control eyes, we subjected mice to a visual cliff task, in order to test general visual function. Figure 6 shows that bilaterally sighted and injected mice differ slightly in their responses to a visual cliff test, but both groups perform significantly above 50% chance levels. Mice were also given a light-dark preference test that revealed no difference between UF008/SAP-injected and control mice with respect to the time spent in the dark or the number of times the mice entered the lighted chamber (65.2±3.6 vs 71.6±5.5% time in the dark and (279±53 vs 185±40 entries into the light for UF008/SAP and controls, respectively; the corresponding values for 9 blind controls were 49.7±4.3 and 316±45, both values significantly different from the other groups, p<.02). When the comparison with controls was limited to the 6 mice that were free-running under LD, there was also no difference between groups.

**Figure 7:**
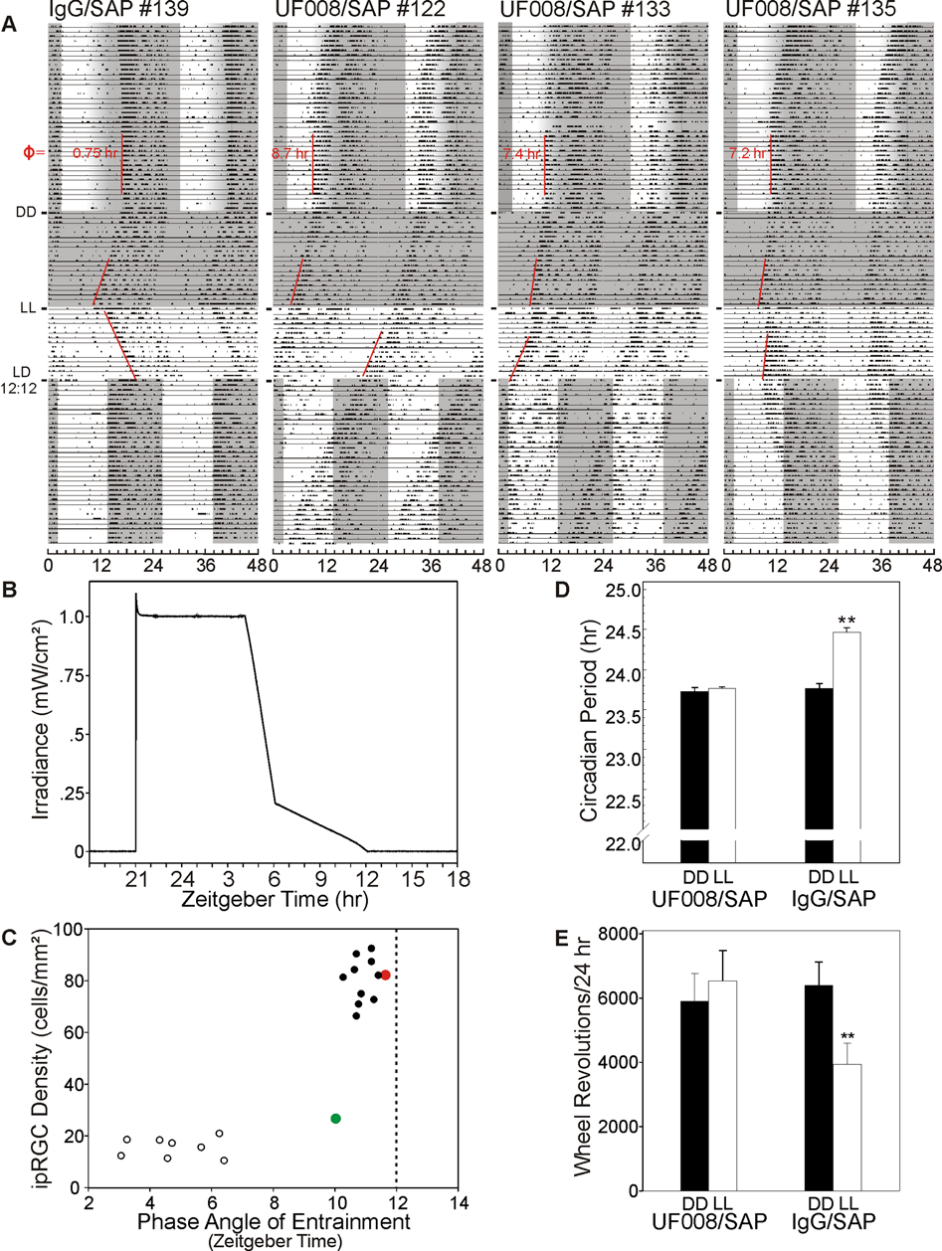
Depletion of ipRGCs greatly alters entrainment, period lengthening in LL and masking by LL. Running records of a control and 3 UF008/SAP-treated mice showing phase angle of entrainment adopted in response to the gradual offset photoperiod, period during DD, during LL and re-entrainment (animals #139, 135) or failure to re-entrain (animals #122, 133) to LD12∶12 (grayed area indicates darkness). The phase angle of entrainment (Φ) during the gradual offset photoperiod is indicated for each individual (ZT12 = light completely off). B) Daily irradiance pattern recorded with a Gigahertz-Optik P-9710-2 universal optometer measured at cage level during the gradual offset light-dark paradigm. C) Relationship between remaining ipRGC density and the stable phase angle of entrainment of adopted by UF008/SAP injected mice and controls during the gradual offset photoperiod. Note the cluster of controls and the outlier animal (green-filled circle), which was injected with the saporin conjugate, but has not lost its ipRGCs. A second outlier animal (red-filled circle) had a normal phase angle of entrainment despite greatly reduced ipRGC density. D) There was no difference in period in circadian period during DD between UF008/SAP and IgG/SAP injected mice, but in LL, IgG/SAP injected mice significantly lengthened their periods (p<0.001; paired t test), becoming significantly different from UF008/SAP injected animals (p<.001, unpaired t test) which did not show any period lengthening in response to LL. E) LL induced masking was absent in UF008/SAP injected mice. Revolutions per day for the last 5 days in DD were compared to those during the initial 5 days of LL. For UF008/SAP mice, revolutions during DD and LL did not differ; for controls, revolutions/24 hr dropped by about 40% (p<.001; paired t tests).

Mice in the rhythm regulation studies were unilaterally injected with 400 ng UF008/SAP or IgG/SAP and contralaterally enucleated (to reduce overall variability in ipRGC ablation) under LD12∶12 conditions before being subjected to various lighting regimes. Figure 7A shows actograms representative of behavior across four different lighting conditions. None of the mice lost entrainment in LD12∶12 as an immediate consequence of UF008/SAP treatment. When switched to a graded photoperiod in which the irradiance gradually declined to zero (LD15G∶9; Figure 7B), all animals entrained, but with markedly different phase angles between groups (Figure 7C). Large differences in phase angle of entrainment were related to the correspondingly large differences in irradiance at the time of activity onset (Figure 7B). In constant darkness (DD), the circadian periods of the two groups did not differ. In constant light (LL), the circadian period of UF008/SAP mice did not change, whereas for control mice the period lengthened, as expected (Figure 7D). When the mice were returned from LL to the original LD12∶12 (without regard to the phase of the individual mice), 9 of 10 UF008/SAP mice required 16 or more days to re-entrain (median = 24 days), whereas every control animal required 14 or fewer days (median = 3 days). Subsequent histology showed that, on the average, UF008/SAP treatment yielded approximately 80% loss of melanopsin cells. Among the UF008/SAP-treated mice, the 3 individuals with the highest densities of remaining ipRGCs required 0, 16 and 16 days to re-entrain (corresponding to 81, 27 and 21 cells/mm^2^, respectively). Four of the UF008/SAP-injected mice never re-entrained. The density of melanopsin expressing RGCs averaged 15.2 cells/mm^2^ in these four animals.

**Figure 8:**
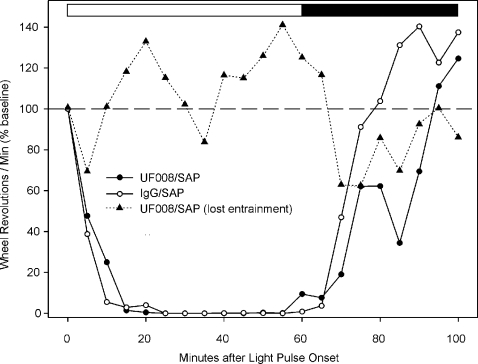
Masking response to a light pulse is absent in mice that lose entrainment to LD12∶12. Masking is nearly normal by UF008/SAP injected mice in response to a 1 hr light pulse. However, the animals recovered more slowly than controls. There is no effect of treatment or of time, but there is a significant interaction (1 way ANOVA with repeated measures, p<.004). In mice that lost entrainment subsequent to UF008/SAP treatment, a 1 hr light pulse failed to induce masking.

Figure 7E shows that constant light exposure induces a suppression of total wheel-running activity (masking) in control (IgG/SAP) mice, but not in UF008/SAP injected mice. Entrained, UF008/SAP-injected mice had relatively normal masking in response to a 1 hr light pulse, but recovered more slowly than expected at the end of the pulse (Figure 8). In contrast, UF008/SAP mice that fail to entrain also do not show masking to a 1 hr light pulse administered about 30 minutes after activity onset.

## Discussion

Saporin is a type I ribosome inactivating protein that was initially identified in the common soapwort plant (*Saponaria officinalis*) [14]. It depurinates a nucleotide in 28S ribosomal RNA resulting in a conformational change that prevents elongation factors from associating with the ribosome, thereby irreversibly arresting protein synthesis at the translocation step. Type I ribosome inactivating proteins lack the lectin-like B chain characteristic of type II ribosome inactivating proteins such as ricin. This peptide facilitates cell entry. Therefore, saporin and other type I ribosome inactivating proteins must be conjugated to moieties that specify their targeting and internalization. Conjugation to ligands of cell surface receptors or to antibodies that recognize cell surface epitopes have been the most common strategies. For example, a substance P/saporin conjugate has been used to specifically ablate inhibitory hippocampal neurons in the rat [15]. 192-IgG/saporin, a conjugate with an antibody specific for the low-affinity neurotrophin receptor, p75^NTR^, has been used extensively to lesion cholinergic neurons in the basal forebrain [16], [17].

The feasibility of using saporin conjugates to ablate specific retinal cell types was demonstrated in rat and ferret [18], [19]. A conjugate consisting of an antibody raised against the vesicular acetylcholine transporter covalently linked to saporin was injected intravitreally. The number of cholinergic amacrine cells in the retina was dramatically reduced.

In this study, we pursued a similar approach to specifically target and kill melanopsin cells. Our saporin conjugate, UF008/SAP, eliminated most of the melanopsin-expressing cells in a dose- and time-dependent manner. Furthermore, the cell killing was uniform across the retina. The percentage of killed cells varied from about 60% to 80% across experiments, and may have depended upon the exact method of injection. The number of remaining cells showing melanopsin immunoreactivity may be an overestimation of functional melanopsin cells as they differed in appearance from those in PBS injected eyes; labeling intensity was reduced in many of the residual cells and the dendrites appeared shorter and broken, suggesting a functionally compromised state.

Other than the loss of melanopsin cells, intravitreal injection of UF008/SAP did not cause any obvious alteration in retinal morphology. The thicknesses of the outer and inner nuclear layers were not diminished compared to controls, suggesting the absence of non-specific cell death. Furthermore, the dendrites arising from the cholinergic amacrine cells retained an appropriate position within the inner plexiform layer suggesting that no drastic reorganization within this synaptic layer had occurred in the face of melanopsin cell loss. Finally, an analysis of visual competency through the use of the visual cliff and light-dark preference tasks indicates that conjugate-injected animals do not suffer any gross defects in visual perception.

Nevertheless, robust effects of UF008/SAP-induced melanopsin cell loss were seen upon examination of certain non-image forming visual responses. These included a greatly impaired ability to entrain properly to the prevailing photoperiod and loss of masking responses to light. In particular, lesioned mice transferred to a “dusk entrainment” paradigm, in which the transition from light to dark occurs gradually, adopted a vastly different phase angle of entrainment. The data support the view that animals with a compromised circadian photosensory system are less sensitive to light as indicated by the fact that they entrain to dusk transitional light only after much longer exposure to much brighter light than necessary for entrainment of control animals injected with a non-specific IgG/SAP conjugate. The number of remaining anti-melanopsin immunoreactive cells was clearly related to the phase angle of entrainment, although the exact relationship is not yet clear.

The absence of a difference in free running periods of the control and UF008/SAP treatment groups in DD suggests that immunotoxin damage does not impact the circadian clock *per se*, but rather exerts its specific actions by modifying photic input pathway to the circadian system. In this respect, the failure of UF008/SAP-treated mice to lengthen their circadian periods in LL is the second important indicator that the circadian visual system is impaired in its response to light. This is in sharp contrast to results obtained from melanopsin null mice which continue to exhibit LL-dependent period lengthening(albeit diminished by about 60%) [20]. The UF008/SAP-injected animals more closely resembled *Opn4^−/−^*; *rd/rd* mice, which are melanopsin-null, lack rod and cone photoreceptors and show no period lengthening under LL. The results indicate that rods and cones require the presence of some minimal number of ipRGCs in order to influence the ability to entrain with a normal phase angle and the period lengthening response to LL.

Light, when administered during an animal's night, acutely suppresses activity, a phenomenon known as “masking” [21]. In addition to its effect on circadian period, LL exposure typically reduces the total amount of nocturnal locomotion. Such activity reduction was observed in control mice, but not in UF008/SAP injected mice. When the UF008/SAP mice were re-exposed to a standard LD12∶12 photoperiod, 5 of 9 failed to re-entrain, a further indicator of diminished circadian system photosensitivity. The locomotor activity of mice that did not entrain animals was also completely unresponsive to a 1 hr light pulse during the early subjective night that produced the expected massive masking in controls. Light pulse-induced masking by the UF008/SAP-treated mice that were entrained was reasonably normal, unlike the LL-induced masking which occurred in all treated mice. This suggests that a greater number of ipRGCs must be lost in order to block re-entrainment and pulse-induced masking than is necessary to block either LL-induced period lengthening or masking.

### Anatomical Considerations

Three separate investigations using different methods have now demonstrated that intact melanopsin cells are necessary in order for the mouse non-image forming visual system to function properly ([22], [23] and this report). Cell ablation greatly reduces the size of the RHT terminal field. In all likelihood, it is this specific change that accounts for the absence of light-induced phase shifts and masking. However, an alternative explanation is possible. Melanopsin cells have dendro-dendritic synaptic contacts with amacrine cells [24], [25] and may transmit photic information to them [26]. Therefore, loss of melanopsin cells might be interrupting necessary transmission of information to the SCN that would pass through amacrine cells in contact with non-melanopsin-containing retinal ganglion cells that also project to the SCN.

The present data (Figure 7C) suggest the possibility that there may be a sharply delineated minimum melanopsin cell density that is required for normal non-image forming visual responses. Potential melanopsin cell photoreceptive network properties may be lost when cell density falls below approximately 25 cells/mm^2^. A similar, sharp threshold effect on rat working memory has been observed following saporin-induced destruction of basal forebrain cholinergic cells [27]. In that instance, memory was impaired only if an approximate 75% cell destruction threshold was exceeded.

The mouse RHT provides bilaterally symmetrical innervation to the SCN [28]. This characteristic has enabled us to compare, in individual mice, the terminal distributions from a normal retina with those from an ipRGC-ablated retina. The results indicate a specialized topography of the RHT terminal field that remains after melanopsin cell cell death. Whether the particular distribution of terminals observed is of functional importance remains to be seen. However, it is not associated with any previously described sector of the mouse SCN based on distributions of peptidergic cell types, clock gene expression or afferent terminal fields.

The thalamic intergeniculate leaflet (IGL) is a component of the circadian system particularly noteworthy because of the robust projection it provides to the SCN. The IGL also receives direct photic input from a predominantly contralateral retinal projection [29]. In mice sustaining UF008/SAP-induced ablation of melanopsin cells, retinal projections to the contralateral IGL are virtually eliminated. This suggests that all or nearly all retinal projections to the contralateral IGL arise from melanopsin cells. A functional implication concerns the role of the IGL as a mediator of circadian system response to tonic light exposure. In the hamster, loss of the IGL reduces the period lengthening effect of LL by approximately the same amount as loss of melanopsin photopigment does in the mouse [20], [30]. Classical photoreceptors account for the remaining effect of LL on period length and may exert their action directly on the SCN via the RHT.

Our results are consistent with those from two earlier studies (Table 1) employing different methods to reach similar conclusions about the essential role played by melanopsin-expressing retinal ganglion cells in the regulation of certain non-image forming visual responses [22], [23]. All three clearly indicate that the absence of melanopsin cells causes a major deficiency of entrainment to LD cycles and a lack of period lengthening in LL. Collectively, the three sets of data demonstrate that melanopsin cells are not necessary for at least rudimentary image forming vision as indicated by response to a wide variety of visual tasks. Unexpectedly, our light-dark preference test, which is unlikely to involve either image formation or optomotor reflexes, was performed equally well by both sighted and melanopsin cell deficient mice, implying that this is behavior relies on different neural mechanisms than used for simple irradiance detection (cf., [31].

**Table 1:** Effect of physical destruction of melanopsin-containing retinal ganglion cells: comparison of results from three studies using different methods.

	Present Data	Reference [23]	Reference [22]
	**METHOD**		
	Anti-Opn4 antibody conjugated to saporin injected in adults	Opn4^aDTA^ mouse	Opn4^Cre/+^;R26^iDTR/+^ mouse adult inducible lesion with diphtheria toxin
**MEASUREMENT**	**RESULT**		
Entrainment	Yes, with advanced onset; about half fail to re-entrain	Most do not entrain, the rest have advanced onset	Absent
Circadian period (DD)	Normal	Longer	Normal
Circadian period (LL)	Same as DD	Shorter than DD	Same as DD
Phase shift to light	–	Absent	–
Masking	Absent^1^	Reduced	Absent
Gross retinal structure	Normal	Normal	Normal
OPN4 cells remaining	18–40%	3–17%	<10%
Residual SCN innervation	Absent centrally; reduced elsewhere	Substantially reduced over 1 yr; more so in homozygotes	Nearly absent
Residual IGL innervation	Nearly absent	Disappears over 1 yr	–
Residual OPT innervation	Robust (CTb)	ipRGC input reduced and disappears over 1 yr	–
Pupillary light reflex	–	Absent at low irradiance^2^	Absent at low irradiance
Visual acuity	–	Slight decrease^3^	
Visual cliff	Normal	–	Normal
Visual learning task	–	Normal	–
Light/dark preference	Normal	–	–
ERGs (scotopic & photopic)	–	Normal	Normal
Optomotor nystagmus	–	Normal	–

1 – but masking was nearly normal in UF008/SAP treated mice that re-entrained to LD12∶12.

2 – at high irradiance, most mice had normal constriction; 3 were reduced by about 50% and these 3 mice failed to re-entrain, unlike the other 6.

3 – possibly attributable to pupil size.

With respect to circadian rhythm regulation, our results are most similar with those of Hatori and colleagues [22] who also used a procedure that acutely killed melanopsin cells in adult mice. In contrast, Guler and colleagues [23] observed effects (longer circadian period in DD, gradual loss of melanopsin cell projections) that are likely the consequence of altered circadian system development. The inducible lesion procedure applicable to adult mice developed by Hatori et al. appears to be the most consistently successful of the three methods used to kill melanopsin cells. In particular, all mice with diphtheria toxin activated melanopsin cell death rapidly lost entrainment and exhibited free-running rhythms. Nevertheless, as is true for the other procedures, this method also fails to kill all melanopsin cells.

The advantages of the UF008/SAP procedure are two-fold. First, we have shown that complete ablation of retinal ganglion cells is not required to observe massive deficits in non-image forming visual behavior of mice, such as circadian rhythm entrainment and masking. Second, this technique has broad potential applicability across species, limited only by the availability of appropriate antibodies that can be conjugated to saporin, creating an melanopsin cell-specific immunotoxin. Finally, this is the first report of a functional immunotoxin made with an antibody to a G protein-coupled receptor. Previous toxins to G protein-coupled receptors have relied on the ligand for targeting and internalization of a toxin [32]–[34]. Antibody targeting of cell-surface proteins has become an effective tool in cancer therapy [35]–[37], and these data indicate that targeting G protein-coupled receptors with antibodies may likewise be useful.

## Methods

### Generation of UF008/SAP immunotoxin

UF008, an anti-melanopsin polyclonal antibody raised in rabbit against the 15 N-terminal extracellular amino acids of mouse melanopsin, was covalently conjugated to saporin (Advanced Targeting Systems, San Diego, California). Synthesis was as described previously [38].

### Validation of UF008/SAP immunotoxin specificity

The RGC-5-mWT-2 cell line was established by stably transfecting RGC-5 cells using calcium phosphate precipitation [39] with a *Sca*I linearized vector containing the *mOpn4* open reading frame (gi∶14349304) in parental vector pcDNA3.1/Zeo(+) (Invitrogen, Carlsbad, CA). Melanopsin expression was confirmed by immunocytochemistry. The RGC-5 cell line was a generous gift of Dr. Neeraj Agarwal [40]. It was derived by transformation of neonatal retinal cells with psi2 E1a, a retroviral vector containing the 12S E1A adenoviral oncogene. RGC-5 cells express mRNA markers characteristic of retinal ganglion cells. Cells were maintained in DMEM (Invitrogen, Carlsbad, CA) supplemented with 10% fetal bovine serum (Atlanta Biologicals, Norcross, GA). Triplicate cultures were exposed to increasing concentrations of the UF008/SAP immunotoxin ranging from 0 to 1000 pg/µl. Controls were exposed to 1000 pg/µl of the nonsense immunoglobulin conjugate IgG/SAP which consists of a general non-specific anti-rabbit IgG covalently conjugated to saporin. In addition, RGC-5 cells not expressing mOpn4 were exposed to 0 or 1000 ng/µl of UF008/SAP or 1000 ng/µl of IgG/SAP. After 4 days in standard culture conditions, representative images of the triplicate cultures were visualized and captured under Hoffman modulation contrast on an Olympus CK2 inverted microscope fitted with a camera mount.

### Animals

C57BL/6J male mice (Jackson Laboratories, Bar Harbor, ME) were used in this study. All the experimental procedures were carried out in accordance with Association for Assessment of Laboratory Animal Care policies and approved by the University of Virginia Animal Care and Use Committee or by the Stony Brook University Institutional Animal Care and Use Committee.

### Eye Injections

Injections (2 µl/eye) were performed in animals anesthetized with isoflurane and eyes topically anesthetized with one drop of 0.5% proparacaine (Akorn Inc, Buffalo Grove, IL). Eyelids were gently retracted with fingers, allowing the eyeball to protrude. A Hamilton syringe with a 30 gauge needle was used to make the injection at the level of the ora serrata into the posterior chamber of the eye and needle was left in place for about 2 minutes after the injections. The lids were then slowly released allowing the eyeball to retract back into the orbit. Animals were systemically administered a mouse ketoprofen analgesic mixture after the injections.

### Dose/response to UF008/SAP

Three month old C57BL/6J male mice were divided into six dose groups (*n* = 5) The right eye of each animal was injected with 2 µl phosphate buffered saline (PBS) vehicle and the left eye was injected with 25, 50, 100, 200, 400 or 800 ng of UF008/SAP suspended in 2 µl PBS vehicle. All animals were sacrificed by CO_2_ asphyxiation 4 weeks post-injection. Eyes from 1 animal per group were sectioned whereas eyes from the remaining 4 animals were used for retinal flat-mounts.

Eyes were excised and hemisected. Posterior eyecups containing the lens were immersed into freshly prepared 4% paraformaldehyde (Electron Microscopy Sciences, Fort Washington, PA) in PBS and kept at 4°C for 24 hours. For flat-mounts, lenses were removed and retinas dissected from eyecups after which they were spread on a filter paper. All immunohistochemical procedures on flatmounts were carried out in 1.5 ml microfuge tubes. For retinal sections, eyecups were cryo-protected in 30% sucrose in 0.1 M PB overnight at 4°C, embedded and frozen in a prepared (3% gelatin, 30% egg albumin in distilled H_2_O) or commercially available (Tissue-Tek OCT 4583 Compound, Sakura Finetek, Torrance, CA) mounting medium, and sectioned in a cryostat at 16 µm thickness. Sections were thaw-mounted onto gelatin/chromium-coated glass microscope slides, air dried, and stored frozen (−20°C or −80°C) until further processing.

Flat-mounted retinas and sections were washed 3 times (10 min each) in tris-buffered saline (TBS) (Quality Biological, Gaithesburg, MD) and blocked for 30 minutes in 6% normal goat serum (Vector Laboratories, Burlingame, CA) in TBS. Blocking was followed by 3 washes with TBS followed by incubation at 4°C for 24 hrs in a 1∶2500 dilution of the primary anti-mouse melanopsin antiserum (UF006) in a TBS buffer containing 1% BSA, 0.25% carrageenan lambda, and 0.3% Triton X-100. Tissues were then washed 3 times with TBS and incubated for 1 hr at room temperature in Cy3-conjugated anti-rabbit IgG secondary antibody (Jackson Immunoresearch, West Grove, PA) diluted 1∶500 in incubating buffer, followed by three TBS washes. Flat mounts were removed from the filter paper and transferred to glass slides. Sections and flat-mounts were mounted in DAPI-containing Vectashield (Vector Laboratories, Burlingame, CA), coverslipped and sealed.

Grayscale images were captured on a Zeiss epifluorescence microscope equipped with a SPOT charge-coupled device camera. Photoshop 6.0 (Adobe Systems, San Jose, CA) was used to enhance image files for brightness and contrast. In each quadrant of the flat-mount, three images corresponding to an area of 0.61 mm^2^ were captured sequentially, from the periphery to the center (optic nerve), for a total of 12 images/retina. These three images were termed “peripheral”, “middle” and “central”. The cell count in each of the 12 frames was converted to cell number/mm^2^. Selected images were pseudocolored with Photoshop to reflect the long wavelength emission fluorescence of the Cy3 fluorophore.

### Time course to UF008/SAP

Three month old C57BL/6J male mice were divided into five groups (*n* = 4). Four groups received bilateral ocular injections of UF008/SAP (400 ng/eye) and were killed 1, 2, 3 or 7.5 weeks post-injection. The fifth group was bilaterally injected with the nonsense immunoglobulin conjugate IgG/SAP as a control and killed at 7.5 weeks. All animals were sacrificed by CO_2_ asphyxiation 4 weeks post-injection. Eyes from 1 animal per group were sectioned whereas eyes from the remaining animals were used for retinal flat-mounts. Tissues were processed as described previously for the dose/response studies.

### Effects of exposure to the immunotoxin on the morphology of the retina

#### Assessment of outer and inner nuclear layer thickness

Retinal sections from the 800 ng/eye dose group of the dose/response study (and the corresponding PBS control) were assessed for the thickness of the outer and inner nuclear layer as indicators of retinal health. Five midsagittal sections containing the optic nerve (or sections within 32 µm of the optic nerve) were analyzed in three non-overlapping microscope fields, according to their eccentricity (“central”, “middle” and “peripheral”). Axiovision Software of the Zeiss epifluorescence microscope was used to measure outer nuclear, inner nuclear and total retinal thickness in each of these areas. The thickness of the nuclear layers were normalized to total retinal thickness at each eccentricity, for both control and UF008/SAP injected eyes.

#### Assessment of retinal architecture

Mice were anesthetized (100 mg/kg ketamine plus 10 mg/kg xylazine, intraperitoneally) and were transcardially perfused with saline followed by 4% paraformaldehyde in 0.1 M phosphate buffer, pH 7.4, with sodium m-periodate (0.01 M) and lysine (0.075 M) added. Eyes were removed, hemisected, and immersed in the same fixative for 1 hour and rinsed with PBS for 30 min. Posterior eyecups were cryoprotected, sectioned, mounted, and stored as described above.

For double-labeling experiments, retinal sections were thawed at room temperature for 20 min., washed three times in PBS, pH 7.4, for 10 minutes, and then blocked in 2% normal donkey serum in PBS for 20 minutes. Tissue sections were incubated for 24 to 48 hours at 4°C in a mixture of a rabbit polyclonal anti-melanopsin antiserum (UF007; 1∶2000 final dilution) and either mouse anti-glial fibrillary acidic protein (GFAP), 1∶500; goat anti-choline acetyltransferase (ChAT), 1∶750 (Chemicon International, Temecula, CA) or mouse anti-calbindin (CALB), 1∶1000 (Sigma-Aldrich, St. Louis, MO). UF007 identifies melanopsin-containing retinal ganglion cells in hamster [41]. About 53% of UF007 positive retinal ganglion cells are β-gal positive in the heterozygous β-gal mouse (Baver, Sollars & Pickard, personal communication), very similar to what is seen using the UF006 antiserum [42]. Primary antibodies were diluted in an incubation solution containing 0.3% Triton X-100 and 5% normal donkey serum. After washing in PBS for 45 minutes and blocking in 2% donkey serum the slides were incubated for 45 min. at 37°C in a mixture of donkey anti-rabbit Texas Red and donkey anti-mouse or anti-goat FITC secondary antibodies (JacksonImmunoResearch Labs, West Grove, PA) diluted 1∶150 in incubation solution. After final washes in PBS sections were coverslipped with ProlongGold (Invitrogen, Carlsbad, CA) and stored at 4°C in the dark.

#### Assessment of retinal projections

Mice (N = 4) were anesthetized with ketamine (100 mg/kg; Wyeth Pharmaceuticals, Madison, NJ) and xylazine (10 mg/kg; Ben Venue Laboratories, Bedford, OH) about 14 days after a unilateral injection of 400 ng UF008/SAP. At this time, the saporin-injected eye received an intravitreal injection of cholera toxin, B subunit, (CT-B) conjugated to Alexa488 and the other eye was injected with CT-B conjugated to Alexa594 (each in 2 µl of 1 µg/µl of CT-B in 0.9% saline with 2% DMSO; both tracers from Molecular Probes, Eugene, OR). After 3 days transport time, the mice were deeply anesthetized with ketamine/xylazine (as above) and perfused with physiological saline and 4% paraformaldehyde in 0.1 M phosphate buffer, pH 7.4. Brains were removed, post-fixed overnight at 4°C, cryoprotected in 20% sucrose for 24–48 hr, then sectioned in the coronal plane at 30 µm on a freezing stage microtome. Four series of free floating adjacent sections were cut from the optic chiasm caudally through the pretectal region and collected in 0.01 M phosphate-buffered saline-azide (pH 7.4). One series was immediately mounted on gelatin-coated glass slides, air dried and coverslipped with Krystalon (Diagnostic Systems, Inc., Gibbstown, NJ).

Photomicrographic false color images were obtained using epifluorescence and a black and white digital camera (Zeiss AxioCam) attached to a Zeiss Axioplan 2 microscope controlled by Zeiss software (Carl Zeiss, Oberkochen, Germany). Images were shot at 10 or 20× magnification, with composites made of the pretectal region. Images were adjusted with Corel Photo-Paint 12 for brightness and contrast and assembled into figures with CorelDraw 12 (Corel Corp., Ottawa, Ontario, Canada).

### Behavioral Experiments

#### Rhythm study 1

For this study of entrainment and period or masking response to constant lighting conditions, each mouse was placed in a cage with a running wheel inside an enclosure in which white LED lighting was computer-controlled. Each enclosure contained five mouse cages with an array (OSRAM OS-CM01E-W) of 9 LEDs above each cage. All arrays per enclosure were controlled via D-A voltage computer output to an LED dimmer (OSRAM OT DIM) with its outputs connected to the arrays. Steady-state maximal irradiance was about 1 uW/cm^2^. Wheel revolutions were monitored by computer, stored as revolutions/min, then summed across each 5 min interval (288/day) and plotted in raster format using custom software (WinCollectRT, Stony Brook University).

When rhythms were stable, each mouse was deeply anesthetized with ketamine and xylazine (as above) and unilaterally enucleated. Under the anesthesia, the remaining eye was injected intravitreally with 400 ng UF008/SAP conjugate (N = 10) or 400 ng rabbit IgG/SAP conjugate (N = 10; 2 additional uninjected mice were included as controls) per 2 µl saline. At the conclusion of the study, the animals were anesthetized, perfused, and their retinas taken for histology using wholemount and cryostat sections.

Prior to the surgery, lighting was LD12∶12, with lights on at 0300 and lights off at 1500. Animals were unilaterally enucleated under LD12∶12, allowed to recover for about 2 weeks, then the remaining eye was injected with 400 ng UF008/SAP or IgG/SAP (2 µL) and animals remained in LD12∶12 for about 2 additional weeks. At this time, because no animals had lost entrainment or adopted a new phase angle of entrainment, all were exposed to a photoperiod in which irradiance gradually declined to zero. This afforded the animals an opportunity to establish a new phase angle indicative of the amount of light exposure necessary to achieve stable entrainment. When entrainment stabilized, each animal was exposed to about 4 weeks DD, followed by 3 weeks in LL, followed by re-exposure to LD12∶12. During LD12∶12, animals were given 1 hr light pulses of 1 uW/cm^2^ white LED light beginning approximately 2 hr after activity onset.

#### Rhythm study 2

Masking in response to a light pulse was tested in mice that either remained entrained to the prevailing photoperiod or lost entrainment as a result of UF008/SAP treatment. In this study, mice were placed in cages with running wheels on shelves in a general animal housing room that provided lighting from overhead fluorescent fixtures producing an LD12∶12 photoperiod and yielding about 20 µW/cm^2^ irradiance at cage level. Mice were allowed to entrain, then anesthetized as described above, unilaterally enucleated and given an injection of 400 ng UF008/SAP or IgG/SAP into the remaining eye. Locomotor rhythms were followed for 2 weeks while the animals remained exposed to LD12∶12 except for a single night-time light exposure for a test of masking. All mice also were subsequently tested for response to a visual cliff and for responses in a light/dark preference test.

#### Masking

Rhythm study 1 demonstrated that UF008/SAP treatment causes a marked drop in sensitivity to light, as measured by the phase angle of entrainment. These mice failed to re-entrain following exposure to constant lighting conditions. In Rhythm study 2, all animals were housed under LD12∶12, but some failed to entrain after simultaneous unilateral enucleation and UF008/SAP injection into the remaining eye. This study of masking was conducted to determine whether the mice that had lost entrainment to light had also masking response to a standard light stimulus. Therefore, a 1 hr light pulse consisting of general room light exposure (20 µW/cm^2^) was initiated at ZT13 for entrained mice or at CT13 to mice that were not entrained. A repeated measures ANOVA was used to compare masking by UF008/SAP and IgG/SAP groups

#### Visual cliff test

Mice were bilaterally injected with UF008/SAP or IgG/SAP (400 ng for each). About 2 wks later, animals were handled daily for several days before beginning the visual cliff test which was conducted in an arena under ordinary laboratory fluorescent light (about 100 µW/cm^2^). The arena consisted of a 59×55×29 cm LWH open-top box with black plastic sides and a 6.4 mm thick clear plastic bottom placed on a desk. Beneath one half of the floor was a black and white checkerboard pattern of 2 cm squares; the other half extended off a desk, about 77 cm above the laboratory floor. Resting on the middle of the floor, dividing the arena into non-cliff (checkerboard) side and cliff side was black plastic beam (2.9 cm high×3.5 cm wide).

Each mouse was lifted by its tail and lowered until all 4 limbs were firmly on the middle part of the beam when it was released. At this moment, the trial was initiated and continued until 5 min had elapsed or the mouse stepped off the center beam with all 4 paws placed one side of the beam. The mouse was then placed back in its cage. The arena walls, floor and beam were cleaned using diluted alcohol on a fresh paper towel between tests. The procedure was repeated ten times for each mouse.

#### Light/dark preference test

The same mice used for the visual cliff test were used for the light/dark preference test begun approximately 2 wks later. The test apparatus consisted of two similar chambers, each about 29×31×30 cm LWH connected by a 5.5 cm square opening. The walls were covered with a pale flooring tile and the floor covered with absorbent paper. A single food pellet was placed in each chamber and a spigot from an externally mounted water bottle protruded into each chamber through the wall opposite the between-chamber opening. One chamber was designated the “dark side” and was covered by an opaque sheet of black plastic. Most of the “light side” was similarly covered by opaque black plastic. However, a central, 11.5×16.5 cm area was removed as a viewport for a Microsoft LifeCam VX-6000 video camera positioned about 11.5 cm above and focused on the chamber floor. The camera was set into, and was surrounded by, opaque black plastic which completed the light side top. Parallel and adjacent to each long side of the viewport, and attached to the underside of the roof, was a Phillips F4T5 Softwhite fluorescent fixture that provided about 100 µW/cm^2^ white light continuously. In the dark side, irradiance was 0.17 µW/cm^2^ directly opposite the between-chamber opening and was darkest (0.04 µW/cm^2^) in the corners nearest the opening.

The camera was set to black and white mode with contrast and brightness adjusted to bleach nearly all visible patterns in the image. Thus, when a pigmented mouse entered the chamber, the number of pixels detected as black per video image increased dramatically (factor of about 50). Custom software detected and counted the number of transitions from dark side to light side and determined the percentage of each minute the animal spent in the light side.

The exposed surfaces of each chamber were sponged clean, washed with water, then dried with paper towels before each test. An animal was placed in the dark side chamber about 1–2 hr prior to lights off in the room where it was normally housed and removed 20–22 hr later. Data collected during the five hour interval beginning at the time of expected activity onset were used for analysis. The number of dark to light side transitions during the 4 hr interval ranged from 68 to 388 (median = 174), indicating that all animals had the opportunity for a large amount of light sampling.
